# Genetic Diversity of Sapoviruses among Inpatients in Germany, 2008−2018

**DOI:** 10.3390/v11080726

**Published:** 2019-08-07

**Authors:** Pia Mann, Corinna Pietsch, Uwe G. Liebert

**Affiliations:** Institute of Virology, Leipzig University, 04103 Leipzig, Germany

**Keywords:** sapovirus, viral gastroenteritis, molecular epidemiology, genotyping, childhood diarrhea, nucleic acid sequencing, phylogenetic analysis, enteric infections, caliciviruses

## Abstract

Sapovirus enteric disease affects people of all ages across the globe, in both sporadic cases and outbreak settings. Sapovirus is seldom assessed in Germany and its epidemiology in the country is essentially unknown. Thus, sapovirus occurrence and genetic diversity were studied by real-time reverse transcription polymerase chain reaction (RT-PCR) and partial sequencing of major viral structural protein (VP1) gene in two different sets of stool samples: (1) a selection of 342 diarrheal stools collected from inpatient children during 2008−2009, and (2) 5555 stool samples collected during 2010–2018 from inpatients of all age groups with gastrointestinal complaints. Results showed year-round circulation of sapoviruses, with peaks during cooler months. In total, 30 samples (8.8%) of the first and 112 samples of the second set of samples (2.0%) were sapovirus positive. Capsid gene sequencing was successful in 134/142 samples (94.4%) and showed circulation of all known human pathogenic genogroups. Genotype GI.1 predominated (31.8%), followed by GII.1 (16.7%), GII.3 (14.5%), GI.2 (13.8%) and GV.1 (12.3%). Additionally, minor circulation of GI.3, GI.6, GII.2, GII.4, GII.6 and GIV.1 was shown. Consequently, sapovirus diagnostics need broadly reactive RT-PCR protocols and should particularly be considered in infants and young children. Further studies from other sampling sites are essential to extend our knowledge on sapovirus epidemiology in Germany.

## 1. Introduction

Throughout the world and among all age groups, human sapovirus infections are associated with acute gastroenteritis, in both sporadic cases and outbreak settings [1,2]. Among patients with sporadic gastroenteritis, sapoviruses were shown to rank second to fourth as the major viral pathogens [2]. In the post-rotavirus vaccine era, their role further increased [3]. Though tending to be somewhat milder, the clinical symptoms of sapovirus gastroenteritis are indistinguishable from those caused by noroviruses [4].

Sapoviruses were first described in 1976 [5] and their name refers to an outbreak in an orphanage in the city of Sapporo in Japan [6]. Along with noroviruses, sapoviruses are classified in the *Caliciviridae* family [7]. The human sapovirus genome is a 7.4–7.5 kb single-stranded, positive-sense RNA molecule with a 3’-end poly(A) tail. The genome commonly encodes two open reading frames (ORFs). Whereas ORF1 translation produces seven non-structural proteins (NS1−NS7) and the major capsid protein VP1, ORF2 is predicted to yield the minor capsid protein VP2 [8].

Based on the VP1 encoding nucleotide sequences, sapoviruses are classified into at least 15 genogroups (GI−GXV) [9], four of which (GI, GII, GIV and GV) are known to infect humans [2]. The human pathogenic sapoviruses in genogroups GI, GII and GV are currently subdivided into seven, eight and two genotypes, respectively (GI.1 to GI.7, GII.1 to GII.8, GV.1 and GV.2) [2,10]. In contrast, the GIV genogroup is less diverse and is thus placed into a single genotype [2].

There have been some long-term studies on the genetic diversity of sporadic human sapoviruses infections. Comprehensive data is available from Japan [11,12]. Recently, studies from Finland [3], South Africa [13], Peru [14] and India [15] substantially increased our knowledge on the global sapovirus genotype distribution.

Sapovirus infections have been reported in stool samples from outbreaks [16] and sporadic cases [17] of gastroenteritis from Germany. Yet unlike norovirus, sapovirus is rarely assessed in diarrhea outbreaks and sporadic sapovirus infections are not notifiable at the national level. Consequently, little is known about sapovirus epidemiology in the country. Thus, the present study aimed to investigate sapovirus occurrence and genetic diversity among inpatients from Germany.

## 2. Materials and Methods

### 2.1. Sample Collection

The presence of sapovirus RNA was assessed in two different sets of stool samples, which had been collected at Leipzig University Hospital, Germany. (1) Retrospective testing was done in 342 stool samples obtained in 2008–2009 from inpatient children with acute diarrhea, who aged less than five years. All the retrospective samples had previously shown negative results for noroviruses, rotaviruses, adenoviruses, astroviruses, enteroviruses and common enteropathogenic bacteria (*Shigella* spp., *Salmonella* spp., *Yersinia enterocolitica* and *Campylobacter jejuni*). (2) During nine consecutive years, 2010–2018, prospective testing was done in stool samples that had been sent to the laboratory for sapovirus routine diagnostics. In total, prospective testing included 5555 inpatients with acute or chronic diarrhea, abdominal pain or any other gastrointestinal complaints. Thereof, 1882 patients aged less than five years and 3673 patients were older. No information upon co-infections was yielded in this set of samples. The male to female ratio was 1:1.3 in both set of samples.

### 2.2. Analysis of Local Temperature

Local temperature datasets were provided by the Leipzig Institute for Meteorology, Faculty of Physics and Earth Sciences, Leipzig University, and were compiled to calculate mean monthly temperatures.

### 2.3. Nucleic Acid Extraction and Sapovirus RNA Detection

Ten-percent stool sample suspensions were prepared in phosphate buffered saline and clarified by centrifugation at 3000 g for ten minutes. Total RNA was extracted (MagNA Pure system, Roche Applied Science, Mannheim, Germany) and RNA aliquots were stored at −80 °C. Presence of human pathogenic GI, GII, GIV and GV sapovirus RNA was assessed by a real-time RT-PCR that target the polymerase–capsid junction region of the viral genome [18]. Briefly, amplicons were generated as recommended by the manufacturer (QuantiFast Probe PCR Kit, OneStep, Qiagen, Hilden, Germany) and detected in real-time by a fluorescent locked nucleic acid probe (5’-FAM-TGGTWYATW+G+G+T+GG-BBQ-3’; TIB Molbiol GmbH, Berlin, Germany) in air-heated glass capillaries (LightCycler 2.0, Roche, Mannheim, Germany). To exclude failure in amplicon detection by probe mismatches, all obtained RT-PCR reaction products were subsequently evaluated by agarose gel electrophoresis.

### 2.4. Nucleic Acid Sequencing

To confirm sapovirus positivity, RT-PCR amplicons of specific size were gel purified (Wizard SV Gel and PCR Clean-Up System, Promega, Mannheim, Germany) and sequenced (BigDye Terminator v1.1 Cycle Sequencing kit and ABI 3500 Genetic Analyzer, PE Applied Biosystems, Foster City, CA, USA) using the consensus reverse primer SaV1245R [18]. In all confirmed sapovirus positive samples, partial ORF1 (806–826 bp) was amplified by real-time PCR forward primers and genotype-specific reverse primers (Table 1) following the manufacturer’s instructions (OneStep RT-PCR Kit, Qiagen, Hilden, Germany).

To enhance genotyping sensitivity, a second-round PCR was performed in samples with low viral load. Forward primer 1245Rfwd (5’-TAGTGTTTGARATGGAGGG-3’) [19] and the genotype-specific reverse primer (Table 1) were used to amplify 710−739 bp of the VP1 gene. If amplification of partial VP1 by genotype-specific reverse primers failed, genogroup-specific reverse primers (SaV-VP1-GI.x-R and SaV-VP1-GII.x-R) were used in a semi-nested RT-PCR in their place.

Amplicons of partial VP1 were gel purified and sequenced (see above) using the applied (semi-nested) PCR forward and reverse primers. Sequence electropherograms were aligned, analyzed and corrected (Geneious v10, Biomatters Ltd., Auckland, New Zealand). The obtained partial VP1 sequences have been deposited in the GenBank database (GenBank accession no. MN164878 to MN165004). More comprehensive nucleotide sequences of seven of the detected sapovirus strains have been published previously (GenBank accession no., MH541021, MH541030, MH541033, MH541039, MH541043, MH763826 and MH763827) [20].

### 2.5. Phylogenetic Analysis and VP1 Genotyping

Partial VP1 nucleic acid sequence alignments (667−697 bp) of the present sapovirus strains and available reference data from GenBank of sufficient length were phylogenetically analyzed using the Maximum Likelihood algorithm in MEGA5 [21]. Statistical support was assessed by bootstrapping with 1000 replicates. Based on the phylogenetic clustering of strains, genotypes were assigned following the proposed genotype nomenclature [2].

### 2.6. Nosocomial Sapovirus Infections

To evaluate the impact of nosocomial outbreaks on the results of genotype distribution and phylogeny of circulating strains, sapovirus infections were classified as community-acquired and nosocomial based on patients’ records review. Infections were classified as nosocomial if the symptoms first appeared 48 or more hours after hospital admission.

### 2.7. Ethical Considerations

This non-interventional study included no additional procedures. Anonymized biological material was obtained only for standard viral diagnosis. Informed consent for storage and further use of samples was obtained from all patients. The study was conducted in accordance with the Declaration of Helsinki, and the protocol was approved by the Ethics Committee of Leipzig University (26 September 2016, AZ 298/16-ek).

## 3. Results

### 3.1. Sapovirus Stool Positivity

Sapovirus RNA was shown in 142 of 5897 stool samples (2.4%). There was no significant difference in sapovirus stool positivity rate between male (2.4%) and female (2.5%) patients. The stool positivity rate was higher in retrospective testing of preselected samples than in prospective testing of unselected samples (Table 2). Samples from children aged six months to two years showed the highest positivity rates in both sets of samples. Lower positivity rates were shown in patients beyond the fourth year of life and in the subset of young infants of less than six months of age in the prospective cohort.

**Figure 1:**
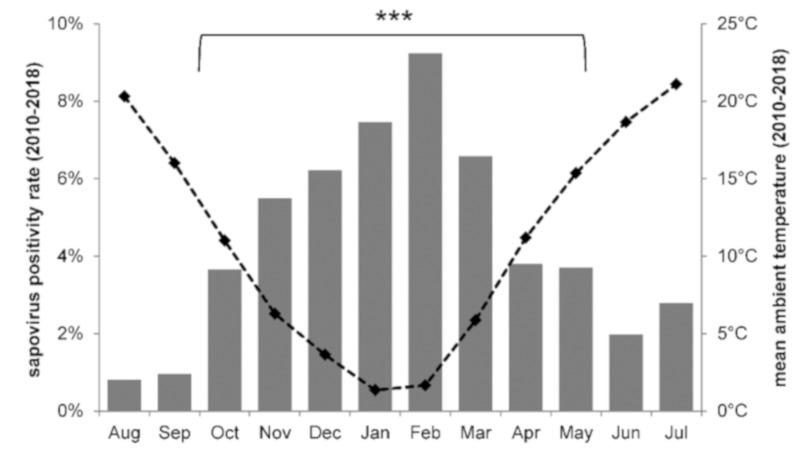
Average sapovirus stool positivity rate per month. Results from samples collected in 2010−2018 from diarrheic inpatients of less than five years of age (*n* = 1882) are shown by grey bars. The dashed black line indicates the local mean temperature per month during the sampling period. The sapovirus stool positivity rate in cold and warm months differed significantly (***, *p* ≤ 0.001).

**Figure 2:**
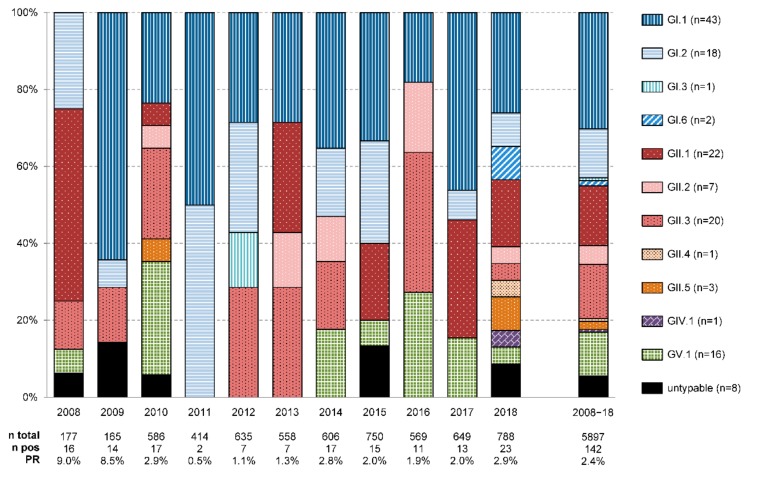
Yearly distribution of sapovirus genotypes detected in stool samples from inpatients in Germany. Additionally, the number of tested samples (*n* total), the number of sapovirus positive samples (*n* pos) and the sapovirus positivity rate (PR) for each sampling year are shown at the bottom. Total numbers per genotype are indicated at the very right. Of note, samples collected in 2008 and 2009 were preselected and restricted to feces from diarrheic under-5 children without enteric co-infections. In contrast, samples from 2010 to 2018 were not preselected and included feces of patients of all age groups.

Sapoviruses were detected throughout the year (Figure 1). In the prospective subset of children within the first four years of life, highest positivity rates were shown from November to March (5.5−9.2%). Samples collected during the cool late fall to spring months were three to four times more likely to be sapovirus positive than those obtained during warmer summer and early fall (odds ratio = 3.67; 95% CI = 1.83−7.37). Sapovirus positivity rates also varied between the different sampling years and ranged from 0.5−2.9% through the years 2010−2018 (Figure 2).

### 3.2. Sapovirus Genotypes

Amplification and nucleic acid sequencing of the partial VP1 gene was successful in 134/142 samples (94.4%). Sapoviruses of all known human pathogenic genogroups were detected (Figure 2). In genotype distribution, GI.1 (30.3%) strains prevailed and were followed by GII.1 (15.5%), GII.3 (14.1%), GI.2 (12.7%) and GV.1 (11.3%) sapoviruses. Minor circulation was shown for GII.2 (4.9%), GII.5 (2.1%), GI.6 (1.4%), GI.3, GII.4 and GIV.1 (0.7% each) genotypes.

Sapovirus genotype distribution fluctuated during the study period (Figure 2). The detected diversity was highest in 2018, when at least ten different sapovirus genotypes were shown in the obtained stool samples. The overall prevailing GI.1 sapoviruses circulated in all years but 2008. In contrast, rare GI.3, GI.6, GII.4, GII.5 and GIV.1 strains were only detected in a single year each.

### 3.3. Sapovirus Phylogenetic Analysis

**Figure 3:**
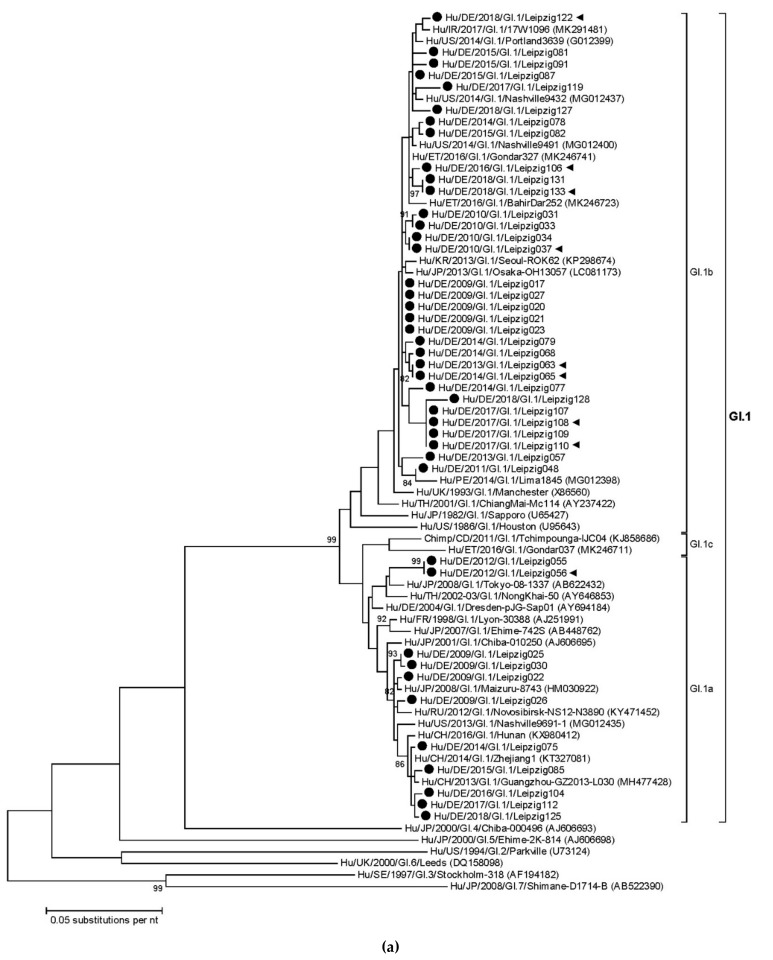

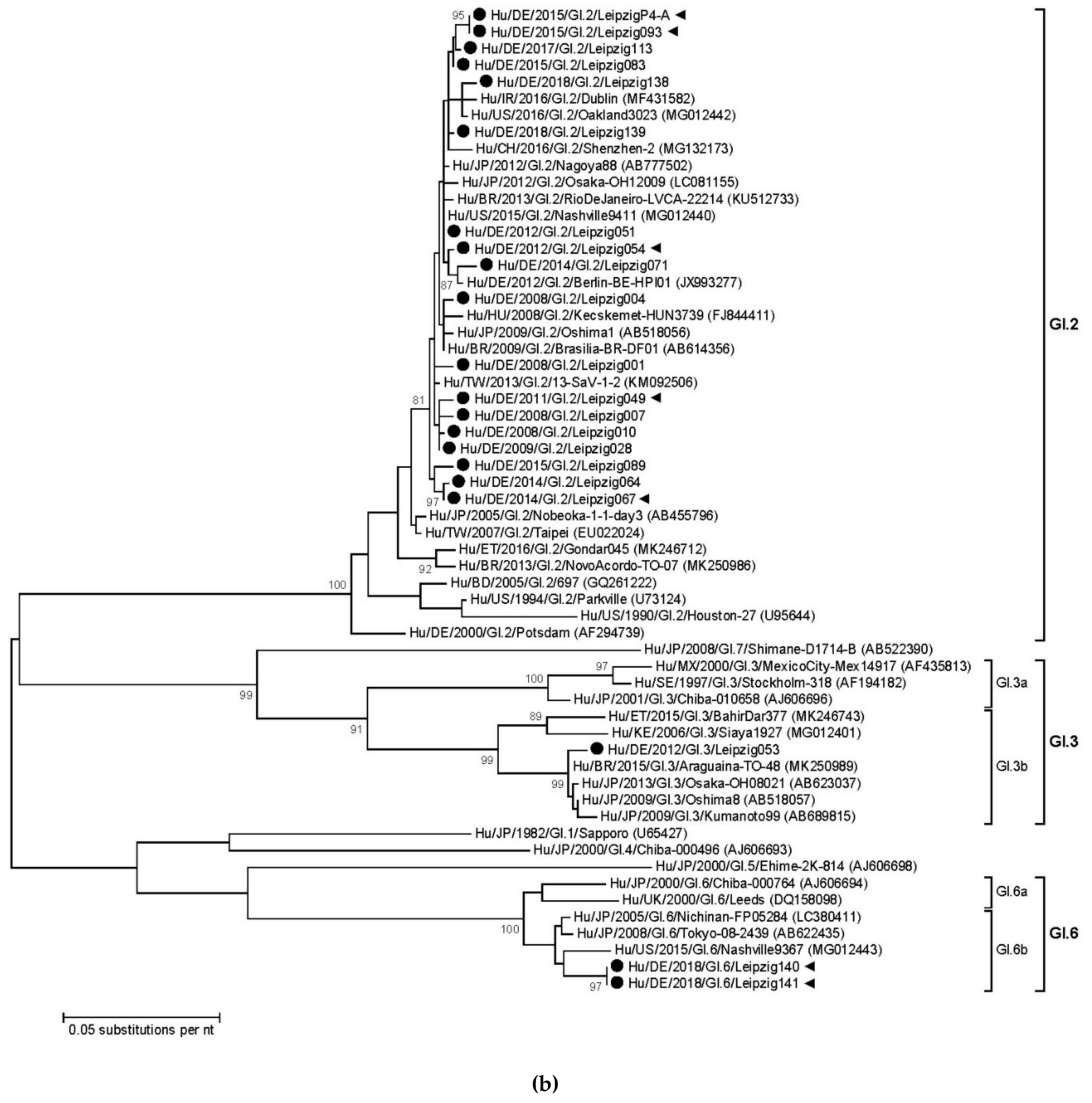

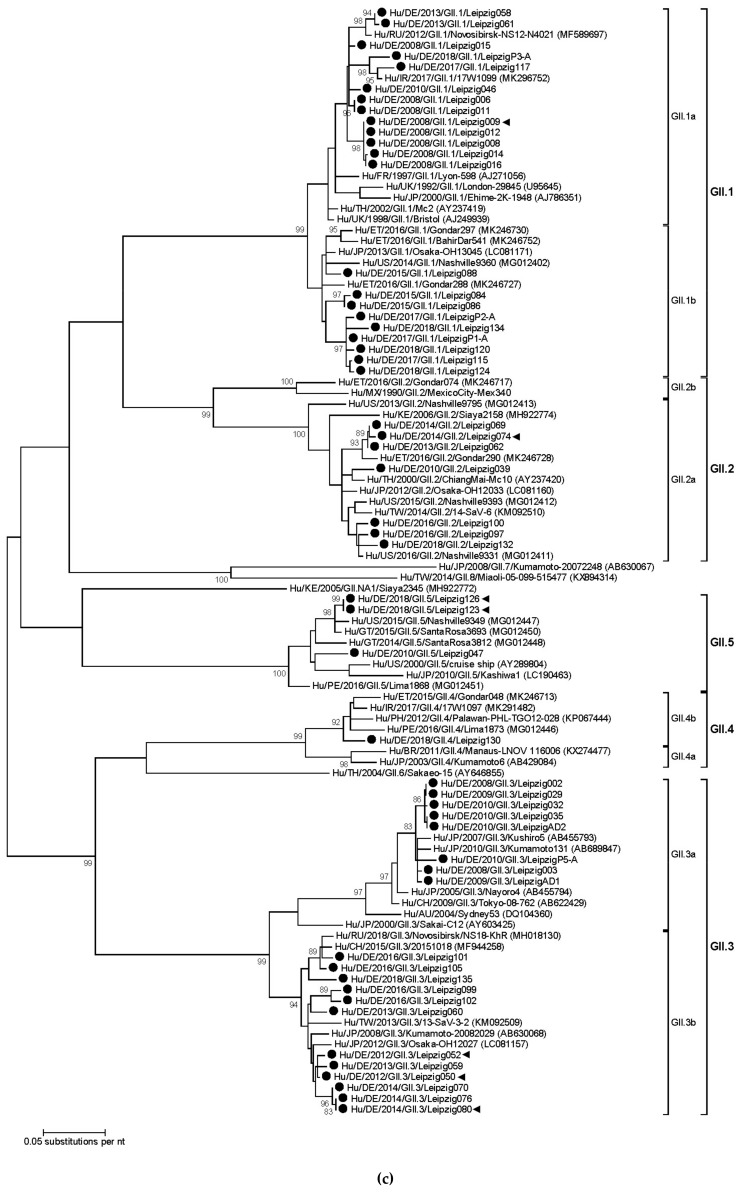

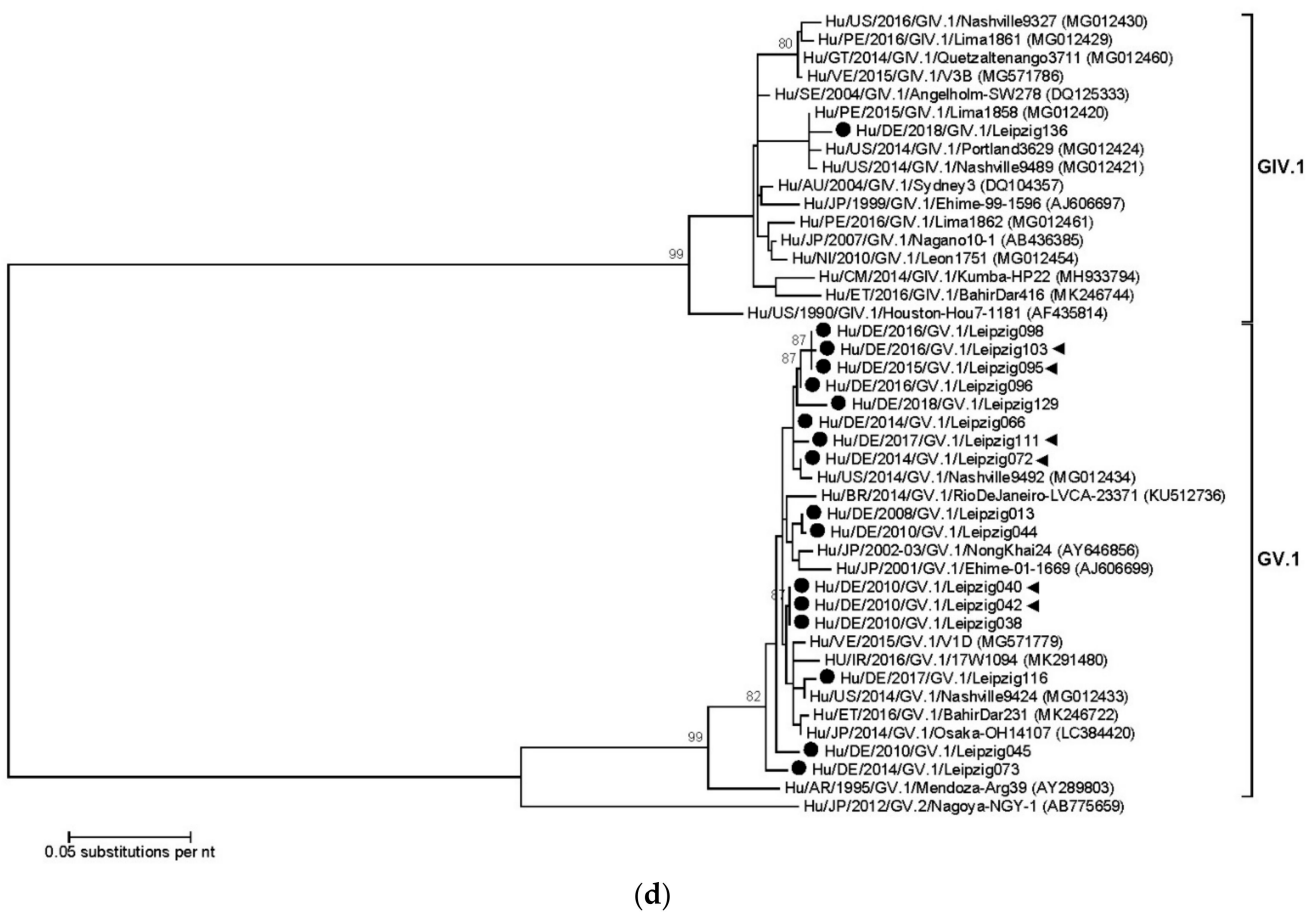
Phylogenetic analysis of partial VP1 sequences (667−697 bp) at the nucleotide level. Separate trees are shown for (**a**) GI.1, (**b**) GI.2, GI.3, GI.4 and GI.6, (**c**) genogroup GII, and (**d**) genogroup GIV and GV sapoviruses. The trees were constructed using the Maximum Likelihood method implemented in MEGA5 software. Bootstraps values (1000 replicates) above 80% are shown. Sapovirus genotypes and intragenotypic lineages are indicated at the right. Strains of the present study are pointed out by black circles. Those detected in nosocomial sapovirus infections are additionally labelled with black triangles.

Within the sapovirus genotypes, variants from different intragenotypic lineages co-circulated (Figure 3). The partial VP1 gene nucleotide identity between the present GI.1 sapoviruses ranged from 94.4% to 100%. Viruses of the GI.1b lineage (*n* = 32) predominated over GI.1a strains (*n* = 11) (Figure 3a). Both intragenotypic lineages were detected in several sampling years. The present GI.1b sequences clustered with reference strains from multiple continents, including sapoviruses from Ireland, the USA, Ethiopia, Korea, Japan and Peru. A similar geographic distribution was shown for related GI.1a reference strains originating from Russia, the USA, China and Japan. For the present GI.1b strain Hu/DE/2018/GI.1/Leipzig128 recent travel history of the patient to Pakistan was recorded. The patient had returned from travel three days before hospital admission.

The nucleotide identity of the present GI.2 strains was high (98.0−100%). They were detected in eight of the eleven sampling years (Figure 2) and all belonged to one big phylogenetic cluster (Figure 3b). The majority of the recent reference sequences were part of the same phylogenetic cluster, too. A GI.3 strain was detected in 2012 and clustered in the intragenotypic lineage GI.3b. It was related to strains from Brazil and Japan, which had been collected during 2009−2015. Identical partial GI.6 VP1 sequences were obtained from two samples collected in 2018 from patients with nosocomial sapovirus infections. They belonged to the intragenotypic lineage GI.6b and clustered with a recent strain reported from the USA in 2015.

In genogroup GII sapoviruses, five different genotypes were detected (Figure 2). The nucleotide identity of partial GII.1 VP1 genes was 91.1−100% and the present strains belonged to two different intragenotypic lineages (Figure 3c). Whereas samples from 2008 to 2018 clustered in lineage GII.1a together with strains from Ireland, Russia and France, lineage GII.1b sequences were only detected since 2015 and showed relatedness to strains from Ethiopia, the USA and Japan. Seven GII.2 strains were detected in the years 2010−2018. Their nucleotide identity was high (94.9−99.9%) and they all clustered in lineage GII.2a with strains from Ethiopia, Thailand, Japan, Taiwan and the USA. For GII.3 sapoviruses, nucleotide identity was lower (85.9%−100%). Whereas all strains from 2008 to 2010 were part of the intragenotypic lineage GII.3a, more recently, GII.3b strains were exclusively detected. The GII.3a strains clustered with reference strains from Japan and China. For GII.3b strains related strains from China, Japan, Taiwan and Russia were available in the GenBank. The present GII.4 sapovirus from 2018 clustered with reference strains from Ireland, the Philippines, Ethiopia and Peru in intragenotypic lineage GII.4b. For GII.5, two different VP1 sequences were shown. One of which was detected in a patient in 2010, the other in two nosocomially infected patients in 2018. Their nucleotide identity was 94.5%. Phylogenetic comparison with the limited number of available reference sequences showed relatedness to strains from the USA and Guatemala.

The genotype GIV.1 sapovirus was shown in one of the present patients. Its sequence from 2018 clustered with strains from the USA (nucleotide identities 98.7−98.8%) and Peru (nucleotide identity 99.1%), collected in 2014 and 2015, respectively (Figure 3d). All the present sixteen GV.1 sequences were highly homologous (97.3−100%), as were the majority of the available global reference sequences (Figure 3d).

### 3.4. Nosocomial Sapovirus Infections

Nosocomial infections occurred in 31/142 sapovirus positive patients (21.8%; 95% CI = 15.8−29.3%). The sapovirus partial VP1 sequences from nosocomial infections clustered in different genotypes, intragenotypic lineages and sub-clusters (Figure 3). In none of the phylogenetic sub-clusters more than two nosocomial cases grouped.

## 4. Discussion

This is the first report to provide comprehensive data on molecular epidemiology of human sapoviruses circulating at a sampling site in Germany. The applied partial VP1 genotyping approach was successful in the vast majority of detected sapoviruses and proved circulation of all known human pathogenic genogroups in the local human population. The genetic diversity of the detected human sapoviruses was high and minor genotypes were present.

In the past, sapovirus GI.1 [16,17,22], GI.2 (GenBank accessions AF294739 and JX993277), GII.1 [16], GII.5 [17] and GIV.1 (GenBank accession AY424804) strains were sporadically observed in isolated human stool samples from Germany. Unfortunately, those sapovirus detections typically provided exclusively short partial polymerase [16] or no sequence data at all [17]. In contrast, the present data is generated from stool samples collected during subsequent years in the same setting and area. Furthermore, sapovirus typing was based on partial VP1 amplification using sensitive genotype specific primers, supplemented with a second-round semi-nested PCR when necessary.

The final phylogenetic analysis was based on sequencing data spanning more than one third of the VP1 gene, which exceeds the coverage of common sapovirus genotyping approaches [3,11,13,14,15,23,24,25,26]. The more comprehensive sequencing data obtained thus allowed marking out intragenotypic lineages and clusters in phylogenetic trees comprising present strains and reference sequence data from GenBank. In this way, local circulation of sapoviruses from distinct intragenotypic lineages was shown for three of the detected genotypes (GI.1, GII.1 and GII.3). In contrast, all present GI.2 strains belonged to a single phylogenetic cluster, which is in agreement with the previously reasoned rapid spread of a novel GI.2 drift variant since 2007 and its subsequent persistence in the human population [27,28].

Consistent with previous studies [3,11,13,14,15,25], sapovirus genotype distribution fluctuated between sampling years. In the whole, GI.1, GII.1 and GI.2 predominated, which have commonly been among the major genotypes in other European countries [3,23,24,25,26,28,29] and beyond [11,14,30,31]. Notably, occasional nosocomial sapovirus infections by various sapoviruses but no major outbreaks were identified in the present patients. Thus, no substantial bias by nosocomial spread towards a specific genotype is assumed for the established genotype distribution.

Phylogenetic relatedness to sapoviruses from several countries and continents was shown for the majority of the present strains. This finding points to a global circulation of sapoviruses and common introductions of new strains into the local population. Obvious vehicles for global virus spread are returning travelers [32]. Such an event may as well explain the unique GI.1 strain in one stool sample detected subsequent to the patient’s visit to Pakistan.

Confirming findings of previous studies conducted in the temperate climate zone [3,23,33,34], sapovirus year-round circulation with peak positivity in late autumn to early spring months was shown. The established stool positivity rates in the range of 0.5−12.8%, depending on preselection of samples and patients’ age, are in line with results from inpatient samples in previous European studies. For instance, sapovirus positivity was 2.4−10.9% in diarrheic feces collected from young inpatients in Italy [24,35] and 6% in inpatient children from the UK [34]. In contrast, recent data from Spain and Denmark point to a possibly higher sapovirus stool positivity rate (15.6−21.6%) among pediatric outpatients and child care center attendees with clinical symptoms of acute gastroenteritis [23,36].

Unfortunately, stool samples of asymptomatic community controls could not be obtained during the study. Thus, based on the data gathered, association of sapovirus presence with clinical symptoms of acute gastroenteritis [36,37,38] could not be thoroughly assessed. Nevertheless, age-stratified sapovirus stool positivity rates were higher in the present retrospective than in the prospective set of samples, whatever the year. Besides sampling years, the inclusion criteria regarding patients’ clinical symptoms represent the major difference between both sets of samples. Whereas the retrospective samples consisted exclusively of feces from children with clinical signs of acute gastroenteritis, the prospective set additionally included samples from patients with chronic diarrhea [20], abdominal pain or any other gastrointestinal complaints.

On the subject of age distribution, the present data shows sapovirus peak positivity during 0.5−2 years of age. Consistently, sapovirus positivity in Danish patients with sporadic gastroenteritis was 3% in the age group of 0−6 months but 12% in older infants and children [25]. The finding of low sapovirus stool positivity during the early months of life was confirmed by other studies [14,15] and suggests a possible protective effect of maternal transplacental and milk antibodies [39,40]. In line with data from Spain and Japan, sapovirus positivity declined again beyond preschool age [23,41]. This observation may be conceivably explained by adaptive sapovirus-specific humoral immunity established during serial sapovirus infection in early childhood [40,42,43,44].

The present study captures sapovirus epidemiology at a single sampling site and is therefore not designed to provide conclusions on the situation all over Germany. It is a well-known fact that occurrence and genetic diversity of sapoviruses may considerably differ between sampling sites [13,30]. Obviously, results are also heavily influenced by the sensitivity and specificity of the applied detection protocols. Though real-time RT-PCR is the gold standard for sapovirus detection [2], its sensitivity is a true challenge in the light of the vast genetic diversity of this viral genus. Continued discoveries of further human pathogenic genotypes [10,45] demand critical review and updates of oligonucleotides for many established sapovirus detection protocols [2,46]. This applies as well to the present one, as comprehensive in silico analysis of currently available sapovirus sequencing data revealed probable detection failure for some human pathogenic genotypes discovered in the meantime, like GII.8 and GV.2 (own unpublished observation). Hence it follows that the established sapovirus positivity rates of the present study have to be considered underestimated. Similarly, the local genetic diversity is presumably higher, even more so as accompanying polymerase sequences would be necessary to assess circulation of sapovirus recombinant strains [47,48].

In conclusion, sapovirus diagnostics should particularly be considered in stool samples from infants and young children with acute gastroenteritis. The genetic diversity of circulating strains is extensive and their genotype distribution varies. Since global spread of emerging sapovirus variants may be fast, novel strains can be rapidly introduced into local settings. Consequently, broadly reactive real-time RT-PCRs should preferably be implemented. Noteworthy, protocols and oligonucleotides may need updates on a regular basis to ensure sustained high sensitivities of the assay. Further studies on more extensive sample sets and from multiple sampling sites are needed and should preferably include outpatients as well as asymptomatic controls to extend our knowledge on sapovirus epidemiology in Germany.

## Figures and Tables

**Table 1:** Reverse primers used in sapovirus VP1 genotyping.

Genotype	Reverse Primer	Sequence (5’→3’)
GI.1	SaV-VP1-GI.1-R	TCTGGTGARACYCCRTTYTCCAT
GI.2	SaV-VP1-GI.2-R	TCAGGTGACACACCATTBTCCAT
GI.3	SaV-VP1-GI.3-R	TCAGGTGACAMYCCRTTYTCCAT
GI.6	SaV-VP1-GI.6-R	TCAGGGGACACACCRTTYTCCAT
GI.x	SaV-VP1-GI.x-R	TCRGGKGAVAHNCCRTTBTSCAT
GII.1	SaV-VP1-GII.1-R	GCRGGTGATATCCCATTGTCCAT
GII.2	SaV-VP1-GII.2-R	GCGGGYGAAATTCCATTGTCCAT
GII.3	SaV-VP1-GII.3-R	GCAGGTGATATGCCRTTRTCCAT
GII.4	SaV-VP1-GII.4-R	GCRGGDGAKAYRCCRTTRTCCAT
GII.5	SaV-VP1-GII.5-R	GCRGGTGATATGCCRTTGTCCAT
GII.x	SaV-VP1-GII.x-R	GCDGGNGANAYNCCRTTRTCCAT
GIV.1	SaV-VP1-GIV.1-R	GCTGGGYGARARCCCRTTCTCCAT
GV.1	SaV-VP1-GV.1-R	GANGGTGARCCTCCRTTCTCCAT

**Table 2:** Sapovirus RNA positivity in stool samples.

Cohort		0 to ≤5 Months	6 Months to ≤2 Years	3 to ≤4 Years	5 to 97 Years
Retrospective 2008−2009	*n*, PR ^1^ 95% CI	4/123, 3.3% (1.3−8.1%)	24/187, 12.8% (8.8−18.4%)	2/32, 6.3% (1.7−20.2%)	n.a.
Prospective 2010−2018	*n*, PR ^1^ 95% CI	3/584, 0.5% (0.2−1.5%)	77/1049, 7.3% (5.9−9.1%)	9/249, 3.6% (1.9−6.7%)	23/3673, 0.6% (0.4−0.9%)

^1^ PR, positivity rate.
